# Microarray Profiling of Phage-Display Selections for Rapid Mapping of Transcription Factor–DNA Interactions

**DOI:** 10.1371/journal.pgen.1000449

**Published:** 2009-04-10

**Authors:** Gordon Freckleton, Soyeon I. Lippman, James R. Broach, Saeed Tavazoie

**Affiliations:** 1 Department of Molecular Biology, Princeton University, Princeton, New Jersey, United States of America; 2 The Lewis-Sigler Institute for Integrative Genomics, Princeton University, Princeton, New Jersey, United States of America; Yale University, United States of America

## Abstract

Modern computational methods are revealing putative transcription-factor (TF) binding sites at an extraordinary rate. However, the major challenge in studying transcriptional networks is to map these regulatory element predictions to the protein transcription factors that bind them. We have developed a microarray-based profiling of phage-display selection (MaPS) strategy that allows rapid and global survey of an organism's proteome for sequence-specific interactions with such putative DNA regulatory elements. Application to a variety of known yeast TF binding sites successfully identified the cognate TF from the background of a complex whole-proteome library. These factors contain DNA-binding domains from diverse families, including Myb, TEA, MADS box, and C2H2 zinc-finger. Using MaPS, we identified Dot6 as a *trans*-active partner of the long-predicted orphan yeast element Polymerase A & C (PAC). MaPS technology should enable rapid and proteome-scale study of bi-molecular interactions within transcriptional networks.

## Author Summary

Specific interactions between protein transcription factors (TFs) and their DNA recognition sites are central to the regulation of gene expression. Inter-species conservation of these TF binding sites (TFBS), and their statistical enrichment in sets of co-expressed genes, facilitates their large-scale prediction through computational sequence analysis. A major challenge in characterizing these putative TFBS is the identification of the proteins that bind them. We have developed a new approach to this problem by expressing random genomically encoded protein fragments as fusions to the capsid of bacteriophage T7. We select this diverse phage-display “library” for binding surface-immobilized instances of the TFBS in the form of short double-stranded DNA. This in vitro selection strategy leads to the enrichment of phage whose capsid-fusion peptides interact with the specific DNA sequence. Because each phage carries the DNA encoding the peptide fusion, the identity of the enriched phage can be determined through population-level PCR amplification of DNA inserts and their hybridization to DNA microarrays. Here, we show that this technology efficiently reveals the identity of proteins that bind known and novel predicted regulatory elements. Its application to a predicted yeast element (PAC) reveals Dot6 as one of its interaction partners, both in vitro and within the yeast nucleus.

## Introduction

The arrival of complete genomes and microarray technology has fueled a revolution in computational predictions of transcriptional regulatory elements, both through inter-species comparative genomics [1],[2] and mapping sequence to gene expression [3]. Application of these approaches to well-studies systems such as *Saccharomyces cerevisiae* has revealed the majority of previously-known TF binding sites, in addition to many novel predictions with strong evidence of function. As the list of high-confidence *cis*-regulatory element predictions grows, a more rapid and efficient approach is needed for the identification of proteins that bind these elements and connect them to the transcriptional regulatory network. Current biochemical and genetic methods of transcription factor identification are laborious and time-consuming. DNA affinity chromatography [4] requires chromatographic experience and biochemical skill, and typically entails several rounds of purification, requiring a significant investment of both time and input protein (due to losses) to isolate a single transcription factor [5]–[9]. Yeast one-hybrid and two-hybrid screens [10] to discover protein-DNA and protein-protein interactions are time-consuming and susceptible to both false positives and false negatives requiring extensive follow-up, especially when transcription factors are the potential interactants [11],[12]. Protein-binding microarrays [13],[14] are dependent upon choosing the right proteins for analysis and the ability to purify a functional epitope-tagged form of those proteins for use as a protein-binding microarray probe.

Phage display has been previously used to study protein-DNA interactions, but this work has focused mainly on the binding of specific zinc fingers to associated DNA nucleotide triplets [15]–[18]. Only a limited number of studies have used phage display libraries to enrich for a natural nucleic acid binding protein by selection against a specific nucleic acid target sequence [19]–[21].

**Figure 1:**
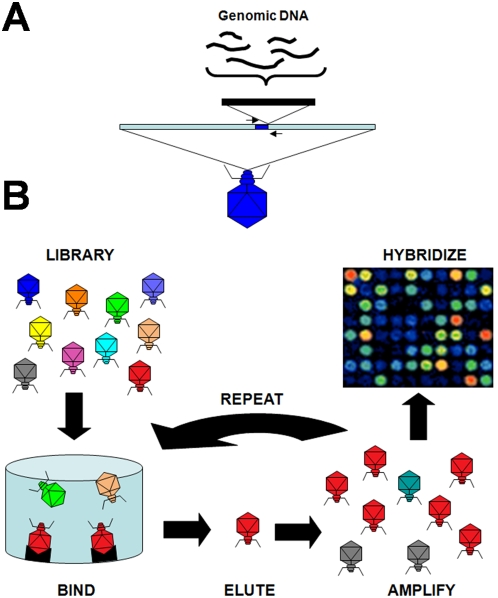
An overview of Microarray profiling of phage-display selection technology. (A) 1–3 kb fragments of yeast genomic DNA are cloned into T7 bacteriophage to create a translational fusion between the capsid protein and the peptide sequence encoded by the insert. (B) The library of phage are exposed to immobilized target DNA molecules and non-binding phage are washed away. Bound phage are eluted, amplified in liquid culture, and the process is repeated over multiple rounds. The sequence content of the enriched phage population is determined by PCR amplification of the inserts, labeling, and hybridization to a yeast ORF microarray.

We have developed a technology for identifying proteins that specifically bind predicted transcriptional regulatory elements (Figure 1). Our approach, called MaPS (for Microarray profiling of Phage-display Selections), selects a diverse (∼10^8^) phage-display library of genomically encoded peptides for binding to surface-immobilized double-stranded DNA containing a DNA motif sequence of interest. After enrichment for a specific DNA-protein interaction, the bound phage are amplified, and can be used for more rounds of selection in order to further enrich the library for specific interactors. Typically, after the appropriate number of rounds of selection, the inserts from the enriched phage can be sequenced individually to identify the interacting proteins. However, in typical selections, the high level of background requires many rounds of phage display enrichment, followed by sequencing of sufficient number of plaques to develop a consensus sequence [22]–[24]. This leads to the selection of phage with only the highest binding affinities at the expense of lower-affinity but biologically relevant interactions. To bypass these limitations, we have developed a simple strategy that effectively ‘sequences’ the entire population of selected phage through PCR-amplification of inserts, labeling and hybridization to a microarray containing all the open reading frames (ORFs) encoded in the genome.

We have chosen a T7 phage system to display peptides between 300–1000 (with a mode of 500) amino acids in length. The T7 phage system offers multiple advantages over other display vehicles, such as Lambda or filamentous phage. The lytic T7 bacteriophage does not have to be exported through the bacterial inner membrane, placing fewer restrictions on the proteins that may be expressed than the more common phage display vector M13 [25],[26]. Other advantages of T7 include extreme robustness to environmental conditions, high capsid-fusion valency (up to 415 per phage), and rapid replication rate.

One of the strongest computationally predicted *cis*-regulatory elements in yeast is the Polymerase A & C (PAC) motif which was initially identified as a conserved sequence found in the upstream region of RNA polymerase I & III subunit genes [27]. Computational analysis of expression data found PAC, in conjunction with the Ribosomal RNA Processing Element (RRPE), to be highly enriched in the upstream regions of a cluster of genes enriched for RNA polymerase I & III transcription, RNA splicing, translation initiation, and other RNA metabolism functions [28]. These sequences are well-conserved among related yeast species [1],[29],[30], and their presence is highly predictive of the expression pattern of their downstream genes [31]. The transcription factor Stb3 was recently identified as the *trans* factor that binds RRPE [32], but to date no PAC-binding protein has been identified, despite numerous attempts [32]–[34].

Here we show that our MaPS technology allows for rapid and proteome-scale survey of sequence-specific protein-DNA interactions. We show that across a variety of test cases, corresponding to known TF-binding sites, MaPS identifies the cognate TF regulators. Moreover, in the most challenging application, we used MaPS to discover the transcription factor that specifically interacts with the PAC element.

## Results

### Phage Display Selection of a Transcription Factor Is Sequence-Specific and Salt-Dependent

In proof-of-principle experiments, the DNA-binding domains (DBD) and complete ORFs of *RAP1* and *MCM1* were cloned into T7 phage and tested for enrichment from the background of a phage display peptide library encoding restriction-digested yeast genomic DNA fragments (see Materials and Methods). Rap1 and Mcm1 were chosen as well-characterized transcription factors whose target genes [35],[36], recognition sequences [5],[37], specific dissociation constants [38],[39], DNA binding domains [40],[41], and crystal structures [42],[43] had been previously determined.

**Figure 2:**
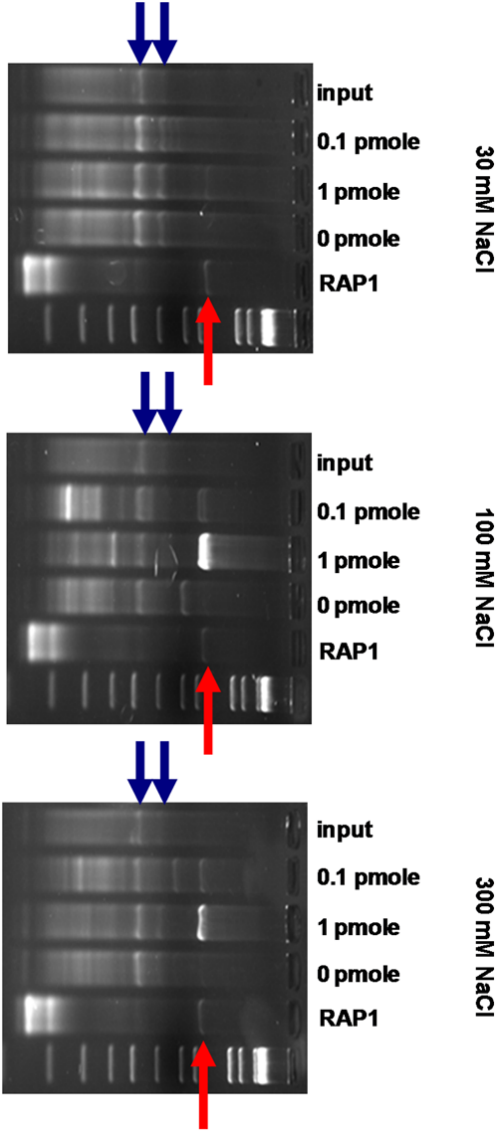
The Rap1 DNA-binding domain is enriched in a sequence-specific and salt-dependent manner. A phage display library was affinity selected against indicated quantities of double-stranded DNA containing Rap1 binding sites under indicated salt conditions. Results from PCR of input phage library (input) or after second round of selection are shown. The intensity of a band is proportional to its abundance in the library. Lanes designated RAP1 indicate results of specific PCR against a single phage with the *RAP1* DNA-binding domain known to be in the library and the red arrows mark the expected size of this clone. Gel-isolation and sequencing of the selected bands at this location confirmed that they correspond to this clone. The blue arrows point out the PCR products corresponding to the *MCM1* DNA-binding domain (lower band) and *MCM1* ORF (upper band). The remaining bands show variable enrichment as a function of salt concentration and likely represent non-specific enrichment during the selection.

A library consisting of T7 phage displaying Rap1 and Mcm1 was mixed on an equal-titer basis with a T7 phage library containing fragments from a yeast genomic DNA complete restriction digest. This library was selected against biotinylated double-stranded oligonucleotides (bRAP1 and bMCM1) consisting of native sequences from upstream of open reading frames *RPS21B* (YJL136C) and *MSG5* (YNL053W) and centered around the pairs of Rap1 and Mcm1 binding sites, respectively. Two rounds of selection were performed against three different amounts of target bDNA in buffers of three different salt concentrations, and the results of the selection determined by parallel PCR-amplification of phage inserts from liquid culture obtained from the second round of selection (Figure 2).

A band corresponding to the *RAP1* DBD clone was present as a result of selection against the oligonucleotide containing Rap1 binding sites (1 pmol) under all three salt conditions. This band was not visible in both the starting library (input) and selections with no oligonucleotide present (0 pmol). The intensity of the band, implying level of enrichment, was dependent upon the amount of oligonucleotide added; the intensity of the band was reduced when only 0.1 pmol of oligonucleotide was used. The band was absent under all three salt conditions when the oligonucleotide selected against contained Mcm1 binding sites instead of Rap1 (data not shown), indicating that the enrichment was sequence-specific, rather than merely due to the presence of double-stranded DNA. No band corresponding to the entire *RAP1* ORF clone was visible in any of the lanes in which a *RAP1* DBD band was present, implying that the complete ORF failed to enrich by selection. This may be due to the under-representation of this clone in the library, due to a bias against large inserts proportional to the size of the translated product [26].

The intensity of the *RAP1* DBD clone bands across the three salt conditions, for a given amount of target oligonucleotide (1 pmol), rose from a minimum in the low-salt condition to a maximum in the intermediate condition, before falling again at the highest salt concentration. This is consistent with greater competition at salt concentrations below physiological levels (100–200 mM NaCl), from phage displaying peptides with nonspecific DNA binding affinity [44]. At salt concentrations above physiological conditions-and approaching that used to elute transcription factors in DNA affinity chromatography [6]-sequence specific affinity is reduced, resulting in washing away of phage bearing sequence-specific interactors.

No band corresponding to either the *MCM1* DNA binding domain clone or to the *MCM1* ORF clone appeared from the selection against the oligonucleotide containing Mcm1 binding sites (data not shown). The bands corresponding to these two clones were visible in the selection against the Rap1 target oligonucleotide (Figure 2), but they appeared at a constant intensity regardless of the amount of DNA present at a given salt concentration, and even at a similar intensity in the starting library (input). We believe that the failure of these doped *MCM1* clones to enrich by DNA affinity selection was due to the close proximity of the Mcm1 DNA-binding domain to the capsid protein. The *RAP1* DBD clone encodes an additional 50 amino acids before the start of the actual binding domain, which may provide flexibility to the domain relative to the phage capsid. On the other hand, only 16 amino acids are encoded between the cloning junction and the MADS box homology region that mediates Mcm1 binding. This is likely insufficient to allow the Mcm1 DNA-binding domain to move into optimum DNA-binding configuration relative to the capsid, as supported by evidence presented later.

### Identification of Known Transcription Factor–DNA Interactions from a Complex Yeast Proteome Library

A diverse phage display library was constructed using yeast genomic DNA partially digested by restriction enzymes that recognize 4 bp restriction sites and leave blunt ends. The library was based on genomic DNA fragments to avoid the bias against low-abundance transcripts in cDNA libraries and the considerable time and labor necessary for constructing a complete ORF library, while at the same time achieving a sub-genic resolution capable of isolating critical domains. The genomic DNA was partially digested with the selected restriction enzymes to produce the greatest possible number of fragments directly ligatable into the T7 genome with no further enzymatic manipulation. The T7 phage genome was altered to accommodate these fragments, adding a 9-glycine linker and a variable 0–2 base pair frame-shift between the capsid protein and the insert site.

**Figure 3:**
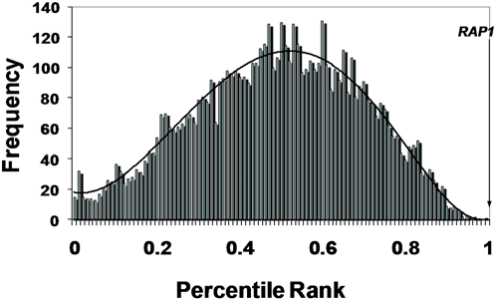
MaPS identifies *RAP1* as the gene whose product interacts with the Rap1 binding site. The yeast genomic DNA phage display library was selected for three rounds against a double-stranded oligonucleotide and PCR products of an upstream region containing Rap1 binding sites. The selected population of phage were profiled through microarray hybridization. Displayed is the distribution of the mean percentile rank for five independent such selections performed. The ORF corresponding to Rap1 had the highest mean percentile rank out of a total of 6242 ORFs queried on the array.

The genomic DNA phage display library was affinity-selected against the oligonucleotide bRAP1, a PCR product containing the sequence from the pair of Rap1 binding sites to the start of *RPS21B* (bRAP1–322), and a PCR product containing the entire upstream region of *RPS21B* (bRAP1–734). The library was selected for three rounds under 100–200 mM NaCl conditions, and PCR monitoring of the liquid lysate demonstrated the enrichment of discrete clones. PCR products were labeled and co-hybridized with a genomic reference. The mean percentile rank values for each ORF were calculated for one selection against bRAP1 and two each against bRAP1–322 and bRAP1–734 (Figure 3), with a high mean rank resulting from consistently high enrichment against all three targets. The ORF with the highest mean rank, and the only ORF >98^th^ percentile, was *RAP1* (YNL216W). The clone corresponding to this insert was also sequenced, and shown to contain a 1-kb fragment of RAP1 that included the DNA-binding domain, ligated in frame with T7 gene 10.

The genomic DNA phage display library was also selected against the oligonucleotide bMCM1 and a PCR product containing the entire upstream region of *MSG5* (bMCM1–401). These targets contained a region with two Mcm1 binding sites flanking a Tec1 binding site [33]. As with the Rap1 binding site, after three rounds of selection, the phage inserts were amplified and profiled through microarray hybridization. The mean percentile rank of the two selections revealed *TEC1* as the most highly ranked ORF and *MCM1* as the 4^th^ most highly ranked ORF. When the clones corresponding to these inserts were sequenced, they were shown to contain in-frame ligations of the entire *MCM1* ORF, and a 1.3-kb fragment of *TEC1* that included the DNA-binding TEA/ATTS domain. Moreover, the *MCM1* clone included the 87 bp immediately upstream of the ATG, resulting in an additional 29 amino acids, plus the 9-glycine linker, between the T7 capsid and the MADS box that mediates DNA binding. This success implies that the direct clone had failed to enrich in the earlier test because the protein was too close to the phage capsid for proper orientation/activity. Most importantly, given that Mcm1 binds DNA as a dimer *in vivo*, its enrichment here clearly demonstrates that dimerization is not an absolute obstacle in the application of MaPS, and that relatively weak, yet specific, protein-DNA interactions can be discovered.

As an additional proof-of-principle, the phage display library was selected against the oligonucleotide bRPN4, derived from upstream sequence of *PRE7* (YBL041W) centered around the Rpn4 binding site, and including a second Rpn4 site in tandem with the native. Three rounds of selection repeatedly resulted in a single clone bearing a 1.5-kb insert (Data not shown). Microarray analysis was not performed because of the absence of other clones and the consistency of the result. Sequence analysis confirmed that the insert contained the 3′ third of *RPN4*, including the region encoding the zinc-finger DNA-binding domain.

### Discovery of the PAC-Element Binding Transcription Factor

After the proof-of-principle validations presented above, we asked whether MaPS was able to discover the novel transcription factor associated with the computationally predicted *cis*-regulatory element PAC. To this end, the phage display library was affinity-selected against bPAC/RRPE, an oligonucleotide centered around the PAC and RRPE sites upstream of *RPC82* (YPR190C), bPAC-320, a PCR product of the entire upstream region of *RPC82*, and bPAC4, a concatemer of four predicted genomic PAC sequences with high computational motif scores, computed using the probabilistic profile captured by the PAC Position Weight Matrix [31]. Three rounds of selection resulted in the enrichment of multiple bands (data not shown). PCR products from two selections against bPAC-320 and one each against bPAC/RRPE and bPAC4 were labeled and co-hybridized to microarrays with a genomic reference, and the mean percentile ranks calculated. The highest percentile rank belonged to YMR130W, an uncharacterized gene with a predicted hydrolase domain, but the second-most highly ranked ORF was *DOT6* (YER088C). We focused on Dot6 as the most likely candidate PAC-binding protein based on previous evidence for a role in transcriptional regulation and because YMR130W also ranked relatively highly in selections for Rap1 (9 percentile) and Mcm1/Tec1 (11 percentile). Isolation and sequencing of the corresponding clone confirmed the in-frame ligation of an 800-bp fragment of *DOT6*, including the DNA binding domain.

Dot6 is a protein with a predicted *myb* DNA binding domain whose binding site could not be identified by chromatin IP [33]. Over-expression of Dot6 reduces silencing at rDNA loci [45], a side effect consistent with the induction of transcription of RNA polymerase I. Both Stb3 and Dot6 have been characterized as binding components of the Rpd3 histone deacetylase complex [46],[47], which has been shown by chromatin IP to bind genes with PAC and RRPE elements in their upstream regions [48].

**Figure 4:**
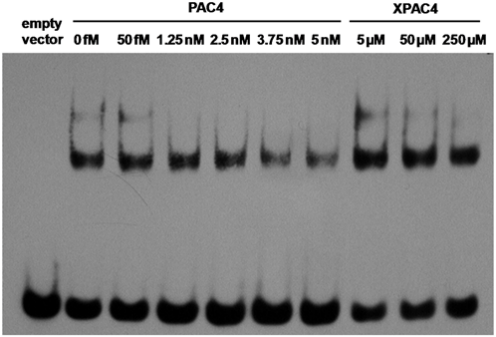
The Dot6 protein is a sequence-specific PAC element binding factor. Gel shift assay was performed with Dot6 DNA-binding domain and DNA containing PAC elements. Purified recombinant GST-Dot6 was incubated with 50 fM biotinylated probe containing 4 copies of the PAC element (bPAC4). Unbiotinylated competitor (PAC4) had identical sequence to the probe, or mutations in each copy of the PAC element (XPAC4).

To establish that Dot6 is indeed a PAC-binding protein, we performed gel shift assays using recombinant Dot6 and oligonucleotides bearing PAC elements. The DNA binding domain of Dot6 was cloned into the pGEX vector and purified by GST tag from *E. coli*, and the purified protein was tested for binding to bPAC4 probe (Figure 4). The probe produced additional shifted bands in the presence of protein purified from the Dot6-expressing strain, but not from the strain containing the empty vector, suggesting that the shift was Dot6-specific. This interaction is sequence specific, being successfully competed by ∼200-fold excess of unlabeled competitor (PAC4), but requiring a ∼10^7^-fold excess of competitor with point mutations in each of the PAC elements (XPAC4).

### Dot6 Binds In Vivo to Promoters with PAC Sites

We have conducted several studies to assess whether Dot6 contributes to regulation of expression in yeast of genes containing PAC motifs. In a series of genetic studies to be reported elsewhere (Lippman and Broach, unpublished observations), we have shown that deletion of *DOT6* has little effect on expression of genes with PAC sites in their promoters during steady-state growth in rich media. Accordingly, Dot6 is not required for expression of such genes under normal growth conditions. However, we observed that Dot6 in conjunction with its paralog Tod6 are required for efficient repression of such genes during nutrient starvation or upon inactivation of the major nutrient responsive signaling pathways, mediated by PKA or TORC1. These studies suggest that Dot6 and Tod6 are redundant repressors of transcription of PAC-containing genes and that they are inactivated by nutrient induced signaling to enhance expression of PAC-site containing genes upon nutrient stimulation.

**Figure 5:**
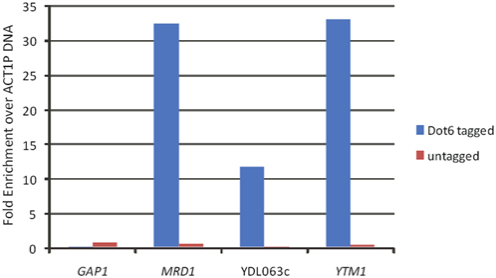
Dot6 binds to PAC-containing promoters in vivo. Chromatin immunoprecipitation was performed on extracts of strain Y3648 (TAP-Dot6) and B4741 (untagged) as described in Materials and Methods. Quantification of these data provided the percent of input DNA for the promoters of each of the indicated genes recovered in the immunoprecipitate. Shown are the fold enrichment of those values relative to the percent input DNA for the *ACT1* promoter recovered in the same immunoprecipitate. PCR quantification was performed in triplicate with less than 20% variation among replicates of individual samples.

In a second series of experiments, we used chromatin immunoprecipitation to examine whether Dot6 binds in vivo to promoters of genes that contain PAC motifs. Given our genetic observations described above, we would anticipate that Dot6 would likely be bound to PAC-containing promoters only under conditions of attenuated nutrient signaling. Accordingly, we assayed for Dot6 DNA binding in cells subjected to carbon starvation, focusing on three genes, YDL063c, *MRD1* and *YTM1*, whose promoters contain 5, 4, and 3 PAC sites, respectively. Transcription of these three genes is substantially repressed upon inhibition of PKA signaling in a *DOT6* strain but this repression is attenuated by at least 3 fold in *dot6Δ* cells (Lippman and Broach, unpublished data). We measured the in vivo association of Dot6 with these promoters, as well as with the promoter of a control gene, *GAP1*, that lacks any PAC sites, by determining the relative amount of promoter DNA immunoprecipitated from a strain expressing a TAP-tagged version of Dot6. These values were then normalized to the relative amount of *ACT1* promoter DNA immunoprecipitated in the same experiment. As shown in Figure 5, promoters for the three genes containing PAC-sites were enriched 12–30 fold over the *ACT1* promoter following immunoprecipitation from the strain expressing TAP tagged Dot6. Significantly less DNA from these promoters was immunoprecipitated from a strain expressing the untagged Dot6 and the small amount precipitated was not enriched relative to the *ACT1* promoter. Finally, the control promoter *GAP1* was not enriched in the immunoprecipitate from the tagged strain. These results are consistent with the conclusion that Dot6 specifically associates with promoters containing PAC sites in vivo.

## Discussion

As both the number of predictions of biologically significant nucleic acid sequences and the number of laboratories making these predictions increases, a rapid and accessible method is needed by which investigators can quickly identify their cognate interacting proteins. The most commonly used approach for identifying nucleic acid-interacting proteins, DNA affinity chromatography, while effective at isolating transcription factors, has considerable up-front costs in material and biochemical expertise not available to most laboratories. Our MaPS technology utilizes phage display, *in vitro* affinity selection, and microarray display in order to rapidly survey the proteome for sequence-specific interactions with a DNA sequence of interest. Another major advantage of MaPS is the ability to simultaneously discover multiple transcription factors that interact with a region of DNA hundreds of base pairs in length. This capability should allow rapid high throughput characterization of the large fraction of non-coding DNA that is under selection for regulatory control. In addition, MaPS allows a laboratory to move quickly and easily from *cis*-regulatory motif prediction to identification of the interacting *trans*-factor without the need for specialized equipment or skills. Where possible, we have made use of commercially available systems and the most common molecular biological techniques in order to maximize the accessibility of the technology.

As with other technologies that are based on molecular libraries of complex genomes, successful identification of transcription factors, using MaPS, relies on high-coverage representation of the coding portion of the genome. We have shown that the use of genomic fragment libraries is a feasible solution in an organism with high gene density (*S. cerevisiae*). However, our success-rate of 70% for identifying known TF-DNA interactions, at least partly, reflects incomplete coverage of the yeast proteome in our phage-display library. The utilization of well-curated ORF collections or normalized cDNA libraries should partly address this challenge in the case of more complex metazoan genomes with much lower gene density. Another challenge is the competition for specific enrichment of TF-DNA interactions by phage whose capsid fusions interact with the solid phase or DNA non-specifically. In addition, some peptide fusions may provide phage with higher reproductive fitness relative to the rest of the library. The exponential amplification of these super-fit phage after every round of selection may also interfere with sequence-specific enrichment of TFs. Experiments presented here show evidence for enrichment of such false-positives. For example, although the highest and the fourth highest ORF signals belonged to the known cognate TFs in the Mcm1/Tec1 selection, the second (*GDS1*) and third (*MET8*) were likely false positives. The gene *GDS1* encodes a mitochondrion localized protein of unknown function and *MET8* encodes a bifunctional dehydrogenase and ferrochelatase involved in seroheme biosynthesis. As would be expected for non-specific enrichment, *GDS1* was also ranked high in the selections against the PAC element (3^rd^ highest) and Rap1 binding sites (112^th^ highest).

The isolation of Dot6 as a PAC element binding protein illustrates the power and unique advantages of our whole-proteome *in vitro* approach. Transcription reporter experiments [49] and association with the Rpd3 histone deacetylase complex [46],[48] imply that Dot6 acts as a repressor of PAC-regulated genes, whose repression is removed under favorable growth conditions. Since both yeast one-hybrid and chromatin IP are normally performed during log-phase growth, the *in vivo* conditions made it unlikely that the Dot6-PAC element interaction could have been detected.

Successive rounds of selection provide us with increased discriminatory power in a manner similar to multiple columns in DNA affinity chromatography. Because the phage are regenerated before each selection round, it is free of material losses typically seen in affinity chromatography. Despite the requirement for multiple rounds, MaPS has rapid turnaround. It was possible to conduct two rounds of selection per day, such that a full PCR readout from three rounds of selection was available by the end of the second day. Because results from the second round were adequate for use on the microarray, it was possible to have fully processed data returned by the third or fourth day. This system is also easily amenable to automation with robotic liquid dispensers and automatic plate washers performing the liquid handling.

## Materials and Methods

### Yeast Strains

Strain Y3648 expressing Dot6-TAP was obtained from Open Biosystems (Huntsville, AL) and an isogenic untagged strain, BY4741, was obtained from Research Genetics.

### Preparation of Recombinant T7 Phage and the Short-Fragment Genomic DNA Library

T7 phage displaying Rap1 or Mcm1 were prepared by PCR amplification and cloning of the yeast genes into the T7 genome. Sequences encoding the DNA-binding domains and complete open reading frames of *RAP1* (amino acids 289–618 and 1–878) and *MCM1* (amino acids 1–135 and 1–286) were amplified from *S. cerevisiae* genomic DNA using primers that added EcoRI sites to the ends of the PCR products. After EcoRI digestion (which truncated the MCM1 ORF to amino acids 1–187 at an internal EcoRI site) the PCR products were mixed on an equimolar basis and cloned into the EcoRI site of the T7Select 10-3b phage vector. Approximately 0.17 µg of ligation product was packaged with an aliquot of T7 packaging extract (Novagen) and amplified in liquid culture as described [50]. Packaging reaction yield and amplified titers were determined by plaque assay as described [50].

A simple library of T7 phage containing short fragments (∼100 bp) of *S. cerevisiae* genomic DNA was prepared as a background from which to select Rap1- and Mcm1-expressing phage. Genomic DNA was completely digested with Tsp509I, which leaves 5′ overhangs compatible with EcoRI. The Tsp509I fragments were cloned into the EcoRI site of 10-3b phage vector, packaged, and amplified in the same way and in parallel with the Rap1 and Mcm1 PCR products above.

### Construction of T7 Phage Containing a Gly_9_ Linker

A short sequence encoding a nine-glycine linker and 0 bp, +1 bp, or +2 bp frameshift was introduced into T7Select 10-3 between the capsid protein gene and the SmaI site. Synthetic oligos encoding a sequence of 9 glycines, followed by 0–2 extra bases, were annealed to their complements to create double-stranded oligos with BamHI and SmaI half-sites at their ends, cloned into the corresponding sites of T7Select 10-3b DNA, and packaged. Samples of individual plaques were resuspended and heated for 10 min at 65°C in 100 µL TE buffer, followed by PCR with primers T7SelectUP and T7SelectDOWN, which flank the T7Select multiple cloning site. Plaques producing PCR products of the correct size were amplified in liquid culture, and successful incorporation of the inserts was confirmed by bidirectional sequencing. Cultures of the correct sequence were stored as T7 strains G9.0, G9.1, and G9.2.

### Construction of Yeast Genomic DNA Libraries

A complex T7 phage display library of the yeast proteome was created from partial restriction digest fragments of *S. cerevisiae* gDNA. Genomic DNA was partially digested with AluI, BstUI, HaeIII, HpyCH4V, or RsaI to produce fragments distributed around a mode of 1.5 KB, and the fragments were size-selected by gel purification to a range of 1–3 KB. Fragments from digestion using each of the restriction enzymes were cloned into the SmaI site of T7 G9.0, T7 G9.1, and T7 G9.2 DNA in separate reactions. 1–4 µg of DNA from each ligation reaction was packaged using T7 packaging extracts, and the number of phage produced by each packaging reaction was estimated by plaque assays. The presence and size of inserts was determined by PCR of random plaques with primers T7SelectUP and T7SelectDOWN. Individual packaging reactions were amplified at a multiplicity of infection of ∼10^−5^ in log-phase *Escherichia coli* BLT5615 culture, made to 0.5 M NaCl after lysis, and clarified by centrifugation. Amplified lysates were pooled to provide the same number of copies of each independent clone from each combination of restriction digest and vector DNA, and the pooled lysate was aliquoted and stored at −80°C. The final library was estimated to contain 6.1×10^7^ independent clones, of which 75% were recombinant, at ∼6500 copies/mL.

### Preparation of Target bDNA

Target double-stranded oligonucleotides containing putative *cis*-regulatory motifs had native sequences from upstream of chosen genes. These sequences were chosen based on the strength of the motif's position weight matrix score and the level of correlation of the associated gene's expression profile to that of the cluster from which the motif was derived [31]. Short (≤70 bp) biotinylated double-stranded target oligonucleotides were prepared by annealing a 5′-biotinylated oligonucleotide to its complementary oligonucleotide in an equimolar ratio. Long (≥200 bp) biotinylated double-stranded DNA targets were prepared by PCR of *S. cerevisiae* ORF upstream regions using one biotinylated and one unbiotinylated primer.

### Phage Selections

Biotinylated target DNA (5 pmol) was bound to the wells of a StreptaWell High Bind strip (Roche) in 200 µL Binding Buffer (30–300 mM NaCl, 20 mM Tris pH 7.5, 2 mM KCl, 1 mM EDTA, 0.15 mg/mL purified BSA (NEB)) and salmon sperm DNA (10 µg, Invitrogen) for 30 min. T7 phage library (3×10^10^ pfu, ∼500 copies/clone) was added to the well and phage-DNA binding permitted for 60 min. Wells were washed five times with 300 µL of Wash Buffer (30–300 mM NaCl, 20 mM Tris pH 7.5, 2 mM KCl, 1 mM EDTA, 0.1% Tween-100) to remove unbound phage. Bound phage were eluted by incubating 30 min in 300 µL of Elution Buffer (1 M NaCl, 20 mM Tris-Cl pH 7.5, 2 mM KCl, 1 mM EDTA). Eluted phage (150 µL) were amplified in BLT5615 log phase culture (5 mL) until lysis (∼2 hr), and the lysate was clarified by centrifugation. Clarified lysate was used as the phage input to the next round, and changes in the phage population due to selection were tracked by PCR of the lysate using the T7SelectUP and T7SelectDOWN primers.

### Microarray Display of Phage Population

DNA was prepared by direct labeling of T7 liquid culture PCR products using Cy3-dUTP (GE Healthcare), Klenow fragment (NEB) and the T7SelectUP and T7SelectDOWN primers, or of yeast genomic DNA using Cy5-dUTP, Klenow fragment and random hexamer mix (GE Healthcare). Labeled probes were purified using the CyScribe GFX Purification Kit (GE Healthcare), aliquots of Cy5-labeled genomic DNA were mixed as a reference with each Cy3-labeled T7 product and concentrated.

Yeast whole-genome spotted ORF microarrays (Microarray Centre, Toronto, ON) were pre-hybridized as described [51], and hybridized as recommended by the vendor [51],[52]. Microarrays were scanned on an Agilent 2565 Microarray Scanner, and the TIFF files processed using GenePix 5.

Median feature and background intensities in both the Cy3 sample channel and Cy5 reference channel for every spot were analyzed by custom Perl scripts. Background-corrected intensities were calculated in each channel for those spots that met minimum signal requirements, with the intensities expressed as a fraction of the total signal intensity in each channel. The final intensity value for each ORF was calculated by averaging the ratio of sample channel to reference channel intensities across the replicate spots for each ORF on each array.

### Gel Shift Assay

Dot6 was isolated by recombinant expression and GST affinity purification. Sequence encoding the Myb homology domain of Dot6 (amino acids 22–278) was amplified from *S. cerevisiae* genomic DNA using primers that added SmaI and NotI sites to the ends of the PCR product. The PCR product was cloned into the corresponding sites of expression vector pGEX4T-3, which provides an N-terminal GST tag, and transformed into *E. coli* strain BL21. Overnight cultures grown in LB broth+50 µg/mL ampicillin were diluted 1∶100 in fresh media, grown to an OD_600_ of 0.5, induced with 1 mM isopropyl-β-D-thiogalactoside, and incubated an additional 1.5 hr. Cells were pelleted by centrifugation and lysed with BugBuster Protein Extraction Reagent (Novagen) with 12.5 µg/mL DNaseI (Roche), 200 µg/mL lysozyme (Roche), and complete protease inhibitor (Roche). GST-Dot6 was purified from the soluble fraction remaining after centrifugation using Microspin GST Purification Columns (Amersham) according to the manufacturer's protocols, eluted with 10 mM glutathione in 50 mM Tris-HCl (pH 8.0), and stored at −20°C.

Purified protein was incubated for 1 hr at room temperature with 25 ng/µL salmon sperm DNA, 0.5 nM target bDNA, and 0–500 µM unbiotinylated competitor DNA in 150 mM NaCl Binding Buffer. The binding reactions were electrophoresed on a 5% polyacrylamide gel, transferred to nylon membrane, and analyzed using a Lightshift Chemiluminescent EMSA Kit (Pierce) according to the manufacturer's protocols.

### Chromatin Immunoprecipitation

We inoculated a 400 ml culture of SC+2% glucose to a density of OD_600_ = 0.12 and grew the cells to OD_600_ = 0.4 at 30°C. Cells were harvested by vacuum filtration and transferred to an equal volume of prewarmed SC media containing no glucose and incubated at 30°C for 80 minutes. Cells were fixed by addition of formaldehyde to a final concentration of 1% and incubated for 20 min at room temperature followed by incubation for 5 min with 0.25 M glycine. Cells were harvested by centrifugation, washed with ice cold PBS buffer, frozen in liquid nitrogen, and stored in −80°C. We resuspended cells from frozen pellets in pre-spheroplasting buffer (100 mM Tris pH 9.0, 10 mM DTT added freshly), incubated the suspension at 10 min at room temperature, harvested cells and resuspended them in spheroplasting buffer (50 mM KH_2_PO_4_/K_2_HPO_4_ pH 7.5, 1.0 M sorbitol, 10 mM DTT added fresh) containing 0.25 mg/ml zymolyase 100T (Seikagaku Corp, Japan). Cells were incubated at 30°C until converted to greater than 95% spheroplasts (ca. 30 min) and then disrupted by vortexing with an equal volume of glass beads. Lysates were sonicated using W-220 Ultrasonics Sonicator at power setting of 2.5 for 8 cycles of 10 sec each. The TAP-tagged protein and associated chromatin were immunoprecipitated using IgG-Sepharose beads (Amersham) overnight at 4°C. The chromatin cross-links were reversed by incubation at 65°C for 6 hrs and precipitated DNA was purified using QIAgen PCR Cleanup Kit (Valencia, CA). Quantitative PCR analysis was conducted using an Applied Biosystems 7900 instrument. Primers for each gene were designed to be less than 100 bp and to encompass all the PAC motifs present in the promoter of the individual gene. The enrichment of occupancy at a gene's promoter was calculated as the ratio of the fraction of input DNA present in the immunoprecipitate relative to the fraction input of *ACT1* promoter DNA present in the same immunoprecipitate.
